# Analysis of the erythropoietin of a Tibetan Plateau schizothoracine fish (*Gymnocypris dobula*) reveals enhanced cytoprotection function in hypoxic environments

**DOI:** 10.1186/s12862-015-0581-0

**Published:** 2016-01-15

**Authors:** Qianghua Xu, Chi Zhang, Dongsheng Zhang, Huapeng Jiang, Sihua Peng, Yang Liu, Kai Zhao, Congcong Wang, Liangbiao Chen

**Affiliations:** Key Laboratory of Sustainable Exploitation of Oceanic Fisheries Resources, Ministry of Education, College of Marine Sciences, Shanghai Ocean University, Shanghai, China; Key Laboratory of Aquaculture Resources and Utilization, Ministry of Education, College of Fisheries and Life Sciences, Shanghai Ocean University, Shanghai, China; Collaborative Innovation Center for Distant-water Fisheries, Shanghai, China; Key Laboratory of Adaptation and Evolution of Plateau Biota, Northwest Institute of Plateau Biology, Chinese Academy of Sciences, Xining, China

**Keywords:** EPO, Qinghai-Tibet Plateau, Schizothoracine fish, Hypoxia adaptation, Positive selection

## Abstract

**Background:**

Erythropoietin (EPO) is a glycoprotein hormone that plays a principal regulatory role in erythropoiesis and initiates cell homeostatic responses to environmental challenges. The Qinghai-Tibet Plateau is a natural laboratory for hypoxia adaptation. *Gymnocypris dobula* is a highly specialized plateau schizothoracine fish that is restricted to > 4500 m high-altitude freshwater rivers and ponds in the Qinghai-Tibet Plateau. The role of EPO in the adaptation of schizothoracine fish to hypoxia is unknown.

**Results:**

The EPO and EPO receptor genes from *G. dobula* and four other schizothoracine fish from various altitudinal habitats were characterized. Schizothoracine EPOs are predicted to possess 2–3 N-glycosylation (NGS) sites, 4–5 casein kinase II phosphorylation (CK2) sites, 1–2 protein kinase C (PKC) phosphorylation sites, and four conserved cysteine residues within four helical domains, with variations in the numbers of NGS and CK2 sites in *G. dobula*. PAML analysis indicated a *d*_N_/*d*_S_ value (ω) = 1.112 in the *G. dobula* lineage, and a few amino acids potentially under lineage-specific positive selection were detected within the *G. dobula* EPO. Similarly, EPO receptors of the two high-altitude schizothoracines (*G. dobula* and *Ptychobarbus kaznakovi*), were found to be statistically on the border of positive selection using the branch-site model (*P*-value = 0.096), and some amino acids located in the ligand-binding domain and the fibronectin type III domain were identified as potentially positive selection sites. Tissue EPO expression profiling based on transcriptome sequencing of three schizothoracines (*G. dobula*, *Schizothorax nukiangensis Tsao*, and *Schizothorax prenanti*) showed significant upregulation of EPO expression in the brain and less significantly in the gill of *G. dobula*. The elevated expression together with the rapid evolution of the EPO gene in *G. dobula* suggested a possible role for EPO in adaptation to hypoxia. To test this hypothesis, *Gd*-EPO and *Sp*-EPO were cloned into an expression vector and transfected into the cultured cell line 293 T. Significantly higher cell viability was observed in cells transfected with *Gd*-EPO than cells harboring *Sp*-EPO when challenged by hypoxia.

**Conclusion:**

The deduced EPO proteins of the schizothoracine fish contain characteristic structures and important domains similar to EPOs from other taxa. The presence of potentially positive selection sites in both EPO and EPOR in *G. dobula* suggest possible adaptive evolution in the ligand-receptor binding activity of the EPO signaling cascade in *G. dobula*. Functional study indicated that the EPO from high-altitude schizothoracine species demonstrated features of hypoxic adaptation by reducing toxic effects or improving cell survival when expressed in cultured cells, providing evidence of molecular adaptation to hypoxic conditions in the Qinghai-Tibet Plateau.

**Electronic supplementary material:**

The online version of this article (doi:10.1186/s12862-015-0581-0) contains supplementary material, which is available to authorized users.

## Background

The Qinghai-Tibet Plateau, referred to as the “Roof of the World” [1], is the highest (approximately 4500 m above sea level on average) and one of the most extensive (2.5 × 10^6^ km^2^) plateaus on earth. The Qinghai-Tibet Plateau possesses an extremely harsh environment characterized by severe hypoxia, severe coldness and strong ultraviolet radiation that has profound effects on animal survival [2]. This unique plateau climate has made the Qinghai-Tibet Plateau a global biodiversity hotspot and a natural laboratory for long-term hypoxia and cold adaptation studies [3–5]. During the evolutionary process, many native organisms developed unique mechanisms to adapt to the harsh plateau environment [3]. Numerous studies have been conducted to explore the adaptive mechanisms of endemic animals. For example, plateau zokor (*Myospalax baileyi*), a subterranean rodent endemic to the Qinghai–Tibet Plateau, has significantly higher microvessel density in cardiac muscle and different structures of hemoglobin and myoglobin compared with other rodents [6]. Mammalian species including Tibetan antelope (*Pantholops hodgsonii*) [7], Plateau pika (*Ochotona curzoniae*) [3, 8], Tibetan wild ass (*Equus kiang*) [9], plateau zokor (*Eospalax baileyi*) [10], and grey wolf (*Canis lupus chanco*) [11] have been intensively investigated. However, few molecular evolutionary studies have focused on plateau fishes.

The plateau schizothoracine fishes (Teleostei: Cyprinidae; also called “mountain carps”) are aquatic vertebrates that are endemic to the Qinghai-Tibet Plateau [12]. The subfamily Schizothoracinae consists of 15 genera containing approximately 100 species [13]. More than 70 species in 12 of these genera are endemic to the Tibetan Plateau [14, 15]. The schizothoracines are among the most diverse subfamilies of cyprinids and are divided into three grades of specialization (primitive, specialized, and highly specialized) according to their habitat elevations and differences in their scales, pharyngeal teeth, and barbels [16]. From the primitive to the highly specialized grade, species show gradual reductions in skin scale coverage, pharyngeal teeth, and barbels with habitat elevation; indeed, the scales of many highly specialized species have been completely lost. Analyses of the mitochondrial genomes demonstrated that there has been positive selection on the protein-coding genes in the mitochondrial genomes of the specialized clade, providing evidence for adaptation to high altitudes in the specialized schizothoracines [12]. For example, Guan *et al*. (2014) revealed that HIF-1αB (hypoxia inducible factor, HIF) might play an important role in the adaptation of schizothoracine fish to the hypoxic Tibetan Plateau [17].

*Gymnocypris dobula*, which has hardly any scales and barbels, belongs to the highly specialized schizothoracines. It occupies the shallow lakes and tributaries at high altitudes (>4500 m) in the Himalayan Mountains, where the low oxygen tension exerts unique selection pressures. *Ptychobarbus kaznakovi* is highly specialized but has a comparably lower altitudinal distribution (3000–4000 m) and was collected to enhance the altitudinal comparisons among species. *Schizothorax nukiangensis Tsao* and *Schizothorax gongshanensis* are specialized schizothoracine fish with a limited distribution throughout Tibet, the upper Nujiang River drainage and the Gongshan area of Yunnan province with a habitat altitude between 1000 m and 3000 m. *Schizothorax prenanti* is a primitive schizothoracine fish with two barbels and small scales covering its entire body that is distributed throughout the upper Yangtze River drainage and upper reaches of the Renhe River in the Hanjiang River drainage, where the water temperature is relatively low (2 °C- 28 °C) [18].

Erythropoietin (EPO) is a glycoprotein hormone that plays a principal regulatory role in erythropoiesis, the process of production of red blood cells [19, 20]. The effects of EPO are mediated by binding to the EPO receptor (EPOR), which is primarily expressed on hematopoietic progenitor cells [21]. EPO:EPOR engagement leads to the dimerization of EPOR and activation of the JAK/STAT signaling pathway [22]. EPO was first isolated from mammals, including humans and mice [23, 24]. It was subsequently identified in teleost species, including the pufferfish (*Fugu rubripes*) [25], zebrafish (*Danio rerio*) [26, 27] and goldfish (*Carassius auratus* L.) [28].

Previous studies showed that hypoxia treatment resulted in increased EPO expression that could mediate the proliferation, differentiation and maturation of erythroid progenitor cells [22, 29]. Hypoxia in mammals and fish has also been reported to induce EPO production through transcriptional regulation by HIF [30, 31]. Despite the low sequence identity between fish and mammals, various studies have suggested conserved functions of EPO/EPOR signal transduction in erythropoiesis in these animals [26, 27]. Additionally, EPO has been reported to initiate survival and proliferative activities in non-erythroid progenitor cells such as endothelial cells, endothelial progenitor cells, cardiomyocytes and neural cells under conditions of moderate environmental stress and tissue damage [32–34]. However, the functions of EPO in the high-altitude adaptation of the schizothoracines to the aquatic fields of the Qinghai-Tibet Plateau remain unknown.

To address this question, the EPO genes from the aforementioned five schizothoracines were characterized. Computational and experimental analyses revealed possibly adaptive amino acid substitutions and an enhanced hypoxic protection function in the EPO gene of the highly specialized *G. dobula*.

## Results

### Characterization of EPO genes from schizothoracines

Samples of *G. dobula*, *P. kaznakovi* (highly specialized schizothoracines), *S. nukiangensis Tsao*, *S. gongshanensis* (specialized schizothoracines) and *S. prenanti* (primitive schizothoracine) were captured from Yadong, the southeast Qinghai-Tibet Plateau, the Qinghai Plateau, the Gongshang and Nujiang rivers in Yunnan Province, and Ya’an in Sichuan Province. Sample information is provided in Table 1.

**Table 1:** Summary information for the five schizothoracine fish samples used in this study

Species	Location	Geographic coordinates	Habitat characteristics		
			Altitude (m)	T(°C)	DO(mg/L)
*G. dobula*	Yadong, Tibet	28°03.37’, 89°17.83’	4506 ± 9	11.0 ± 0.2	1.9 ± 0.3
*P. kaznakovi*	Xialaxiu, Qinghai	32°38.13’, 96°33.8’	3911 ± 9	12.0 ± 0.2	3.6 ± 0.3
*S. nukiangensis Tsao*	Nujiang, Yunnan	25°41.24’, 98°53.22’	1201 ± 7	16.0 ± 0.2	7.8 ± 0.5
*S. gongshanensis*	Gongshan, Yunnan	27°39.23’, 98°43.12’	1212 ± 9	15.0 ± 0.3	7.7 ± 0.4
*S. prenanti*	Ya’an, Sichuan	29°98.48’, 103°01.19’	950 ± 3	18.0 ± 0.2	9.0 ± 0.5

*T* temperature under the water (°C); *DO* dissolved oxygen. Habitat characteristics data were obtained from Global Position System and YSI water quality analyzer (Xylem, America)

**Fig. 1:**
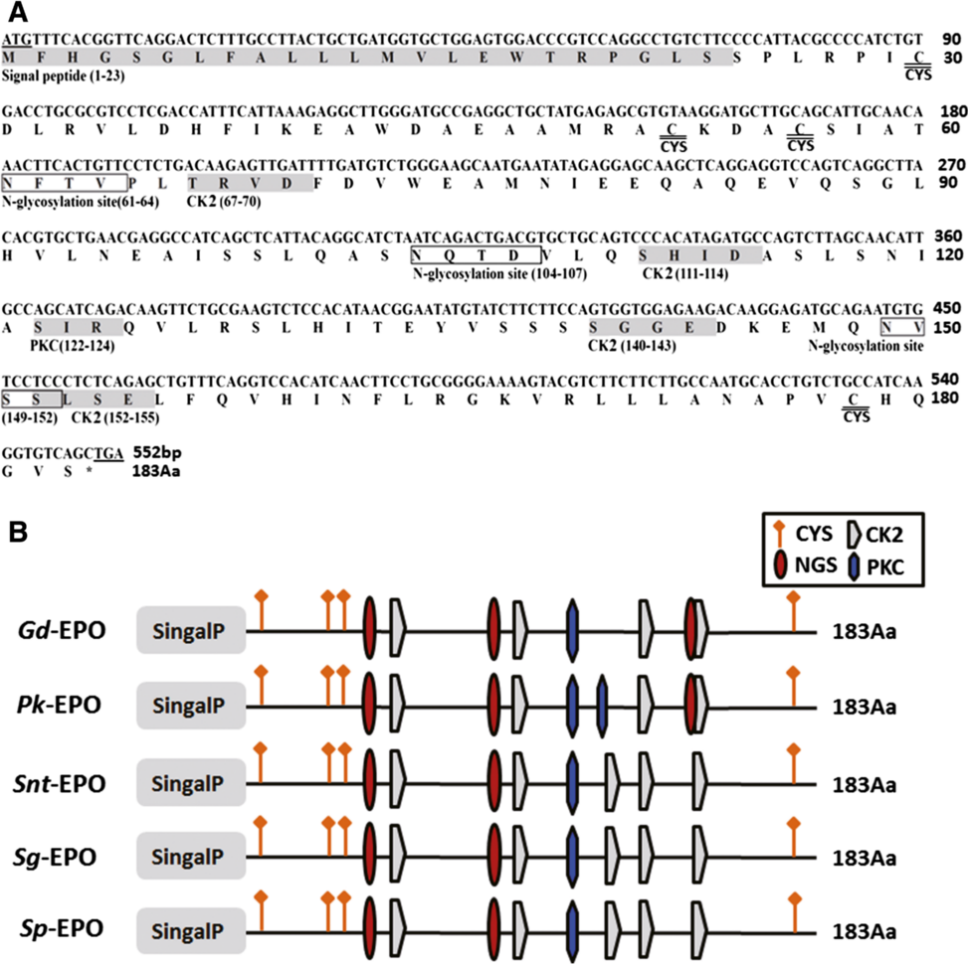
The sequences of *G. dobula* EPO and the schematic representation of the five schizothoracine fish EPOs. **a** *G. dobula* (*Gd*) EPO mRNA and deduced amino acid sequence. The predicted motifs and signal peptide sequence are shaded with a gray background with name abbreviations. The boxed amino acid sequences indicate the N-glycosylation motif sites. The start codon (ATG) and the stop codon (TGA) are underlined. The asterisk in the amino acid sequence indicates the stop codon. The motif abbreviations are presented as follows: CK2, casein kinase II phosphorylation site; PKC, protein kinase C phosphorylation site; CYS, cysteine. The nucleotide and deduced amino acid residues are numbered on the right. **b** Schematic representation of the architecture of the five schizothoracine fish EPOs. The structural domains are marked by different box patterns as indicated in the legend. The length of each bar is proportional to the size of the represented domain. The number of amino acids is shown to the right of each EPO

The coding regions of the EPO full-length cDNAs for the five species were cloned and sequenced. The full-length coding sequences from the translation start codon (ATG) to the stop codon (TGA) were 552 bp in length in all five species and encoded EPO proteins of 183 amino acids (Fig. 1).

Sequence analysis indicated that the deduced amino acid sequence of schizothoracine EPO included a signal peptide predicted to be cleaved between amino acids (AAs) 23 and 24 (Fig. 1). The predicted motifs included one protein kinase C (PKC) phosphorylation site, 4 casein kinase II (CK2) phosphorylation sites, 2 N-glycosylation (NGS) sites and four conserved cysteine (CYS) residues (Fig. 1a). An additional NGS (149–152) and the loss of a CK2 site were uniquely found in the high-altitude schizothoracine *G. dobula* and *P. kaznakovi* EPO structures, which differed from lower-altitude EPOs (Fig. 1b).

### Phylogenetic analysis of the schizothoracine EPO protein

**Fig. 2:**
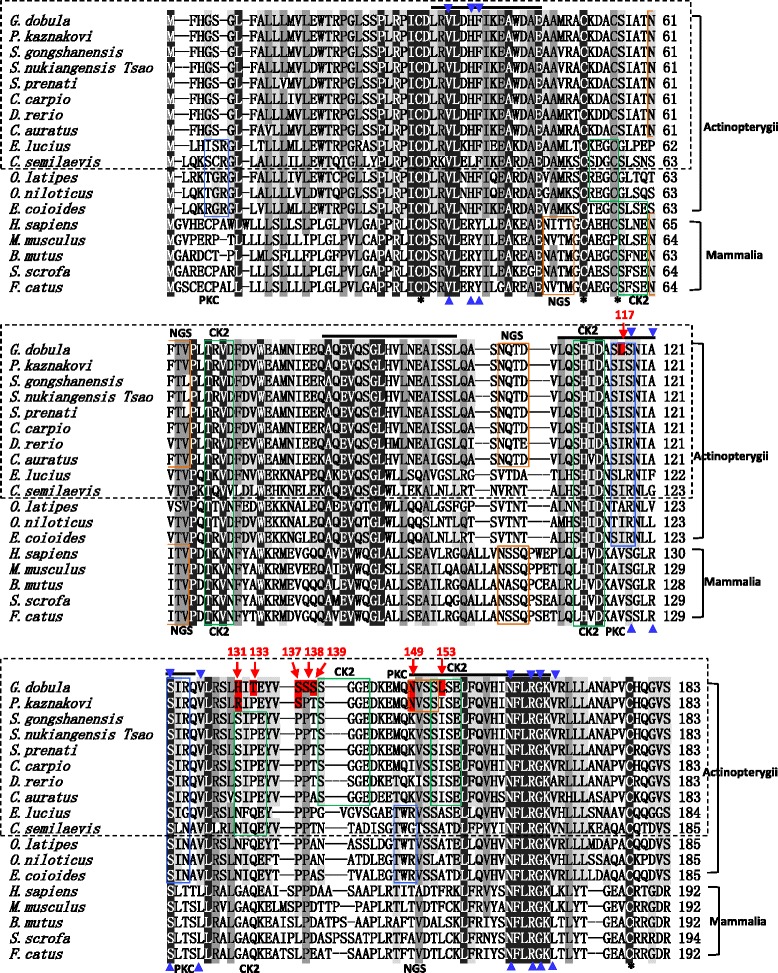
Sequence alignment of the EPOs. Seventeen EPO sequences, including those of 13 Actinopterygii fish species [*G. dobula* (KT188754), *P. kaznakovi* (KT188755), *S. nukiangensis Tsao* (KT188756), *S. gongshanensis* (KT188757), *S. prenanti* (KT188758), *C. auratus* (AGH20610), *C. carpio* (ABB83930), *D. rerio* (AAI62974), *E. lucius* (XP_010901758), *C. semilaevis* (XP_008325395), *O. latipes* (XP_004079700), *O. niloticus* (XP_003457688) and *E. coioides* (AAW29029)] and 5 mammals [*S. scrofa* (CAB96416), *F. catus* (AAA18282), *B. mutus* (XP_005910850), *M. musculus* (AAI44884) and *H. sapiens* (CAA26095)] were aligned with ClustalW. The ten species used for the phylogenetic tree construction (Fig. 3) are highlighted in a dashed rectangle. The limits of each helix are drawn according to the human EPO protein sequence and marked by bold lines. The four conserved cysteine residues are marked by asterisks. The NGS sites, the CK2 phosphorylation sites and the PKC phosphorylation sites are marked with orange, green and blue rectangles, respectively. The EPO receptor binding sites were inferred based on a structural model of human EPO [35] and are indicated by blue triangles. The deduced amino acid residues are numbered on the right. The amino acid sites unique to the two highly specialized schizothoracine fish *G. dobula* and *P. kaznakovi* within the Cyprinidae fishes are highlighted with red shadows and numbers

To investigate the evolutionary relationships between the vertebrate EPOs, we gathered the EPO protein sequences from the newly characterized schizothoracines, goldfish (*Carassius auratus*, AGH20610), carp (*Cyprinus carpio*, ABB83930), zebrafish (*Danio rerio*, AAI62974), northern pike (*Esox lucius*, XP_010901758), tongue sole (*Cynoglossus semilaevis*, XP_008325395), medaka (*Oryzias latipes*, XP_004079700), tilapia (*Oreochromis niloticus*, XP_003457688), orange-spotted grouper (*Epinephelus coioides*, AAW29029), 2 amphibian EPO sequences (western clawed frog (*Xenopus tropicalis*, ADJ68000) and African clawed frog (*Xenopus laevis*, BAI82351)) and 6 mammalian EPOs (dog (*Canis lupus familiaris*, AAS77874), pig (*Sus scrofa*, CAB96416), cat (*Felis catus*, AAA18282), yak (*Bos mutus*, XP_005910850), mouse (*Mus musculus*, AAI44884) and human (*Homo sapiens*, CAA26095)). A multiple sequence alignment of the 13 Actinopterygii and 5 mammalian EPO proteins is shown in Fig. 2.

The sequence alignment indicated that all vertebrate EPOs possessed NGS, PKC phosphorylation sites, CK2 phosphorylation sites, four conserved cysteine residues and four predicted helical domains (Fig. 2). Although the overall sequence similarities between Actinopterygii and mammalian EPOs were low, the NFLRGK sequence in the fourth helical domain was conserved among these lineages (Fig. 2). The residues potentially involved in EPOR binding (marked with blue triangles in Fig. 2) were identified based on a structural model of the human EPO [35]. It was clear that the EPOs of all eight Cyprinidae fish shared high sequence similarities (91.8–99.5 %) and contained similar motifs (Fig. 2). Within the amino acids which are diverged between the high-altitudinal and the rest of Cyprinidae fish, two amino acid sites (137S and 149 N) were shared by the high-altitudinal schizothoracines *G. dobula* and *P. kaznakovi*, and six were unique (117 L, 131H, 133 T, 138S, 139S, and 153 L) to the *G. dobula* EPO gene (Fig. 2). Although an additional round of whole-genome duplication occurred during teleost evolution compared to other vertebrate lineages, the EPO gene remained as a single-copy gene in all of the teleosts examined.

### Test for positive selection in *G. dobula* EPO and EPOR gene

**Fig. 3:**
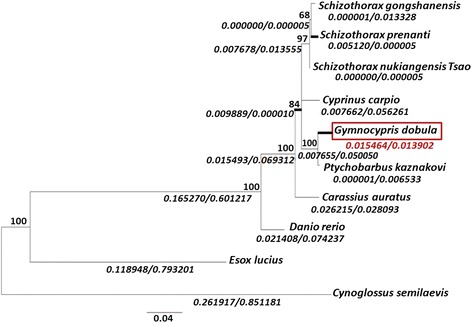
Evolutionary relationships of the EPOs. The phylogenetic tree was constructed using the ML method, as described in the Methods. The calculated *d*_N_/*d*_S_ (ω) values (in italics) and bootstrap values are shown in each branch. The branches with ω values ≥ 1.0 are shown in bold. The Tibetan Plateau schizothoracine *G. dobula* assigned to the foreground is highlighted by a red solid rectangle

Given the unusual amino acid substitution pattern detected in *G. dobula* EPO, we investigated whether it has undergone adaptive evolution. We obtained the EPO coding sequences from five schizothoracine fish (*G. dobula*, *P. kaznakovi*, *S. nukiangensis Tsao*, *S. gongshanensis* and *S. prenanti*) and five other fishes (*C. auratus*, *C. carpio*, *D. rerio*, *E. lucius* and *C. semilaevis*). These species were chosen for analysis because they either belong to the clade Cyprinidae or are most closely related to the EPOs of the schizothoracines currently available from the public database. Codon substitution analysis was performed using the PAML 4.8 software [36] based on the phylogenetic trees generated using the maximum likelihood method (Fig. 3).

First, we estimated the relative evolutionary rate using the free-ratio model, which assumes an independent *d*_N_/*d*_S_ (ω) ratio for each branch of the tree. As shown in Fig. 3, the branch of *G. dobula* demonstrated a *d*_N_/*d*_S_ (ω) value of 1.112 (Fig. 3). The *d*_*N*_ (0.015464) and *d*_*S*_ (0.013902) calculated from *G. dobula* EPO were both significantly higher than those of EPOs from other schizothoracines (Fig. 3), indicating a possibly accelerated evolution in this branch among the schizothoracines.

The tree length based on *d*_*S*_ is approximately 2.402 according to the estimation by the M0 model. The divergence is sufficient to employ the branch-site model to evaluate branch-specific sites for positive selection. Under this model, we assigned *G. dobula* as the foreground branch, and all other branches were set as the background branches. Although the LRT (likelihood ratio test) was not significant, five sites (117 L, 131H, 133 T, 138S and 153 L) were identified as potentially under positive selection, with the posterior possibilities ranging from 0.571 to 0.797 based on Bayes Empirical Bayes estimation (Table 2). Of these sites, 131H was unique to the two high-altitude schizothoracines (*G. dobula* and *P. kaznakovi*), and 133 T, 138S, and 153 L were unique to *G. dobula* (Fig. 2).

**Table 2:** Parameter estimates for the evolutionary analysis of the schizothoracine EPO

Models	Estimate of parameters	ℓ	Positively selected sites	*P* value
Branch model				
free-ratio	(see Fig. 3 for ω values for each node)	−2272.00	None	
Branch-site models (LRT for branch-site: 0.20)				
Model Null	*p*0 = 0.19, *p*1 = 0.08 (*p*2 + *p*3 = 0.73), ω0 = 0.14, ω1 = 1.00, ω3 = 1.00	−2267.82	None	
Model A	*p*0 = 0.48, *p*1 = 0.21(*p*2 + *p*3 = 0.31), ω0 = 0.14, ω1 = 1.00, ω3 = 2.45	−2267.72	117 L (*p* = 0.727), 131H (*p* = 0.621)	0.6547
			133 T (*p* = 0.719), 138S (*p* = 0.797)	(df = 1)
			153 L (*p* = 0.571)	

The branch-site model in codeml program divides all the sites into four classes, class 0, 1, 2, and 3. Class 0 is for the sites under purifying selection. Class 1 is for the sites under neutral selection. Class 2 and 3 are for the sites that have positive selection in foreground branches. In this table, *p*_0_ and ω_0_ stand for the percentage and averaged omega value of class 0 sites in the alignment. *P*_1_ and ω_1_ stand for the percentage and averaged omega value of class 1 sites. *P*_2_ + *p*_3_ and ω_2_ + ω_3_ are for the percentage and averaged omega value of the sites in class 2 and 3

Similarly, EPOR coding sequences from the same ten fish species were obtained and used for positive selection analysis. The multiple sequence alignment of the EPOR proteins is shown in Additional file 1: Figure S1. According to PAML analysis, the ω (*d*_N_/*d*_S_) value of the *G. dobula* EPOR branch was <1 (Additional file 2: Figure S2), and that of the branch leading to the two high-altitude schizothoracines (*G. dobula* and *P. Kaznakovi*) EPORs was close to 1 (0.029107/0.030475 = 0.9551), greater than those found in other branches (Additional file 2: Figure S2), suggesting the possible acceleration of amino acid substitution in this branch. Some amino acids were predicted under potentially positive selection using the branch-site model by assigning the two high-altitude schizothoracines as the foreground and all other branches as the background branches (Additional file 3: Table S1). Most of the identified positive selection sites (15 out of 19) were located in the ligand-binding domain and the fibronectin type III domain (Additional file 1: Figure S1) of the EPOR protein. However, the LRT results for the branch-site model for both EPO (*P* = 0.2) and EPOR (*P* = 0.09) genes fell short of being statistically significant (Table 2 and Additional file 3: Table S1), suggesting the selection on the two genes were statistically weak.

### Structural prediction of the *G. dobula* EPO

**Fig. 4:**
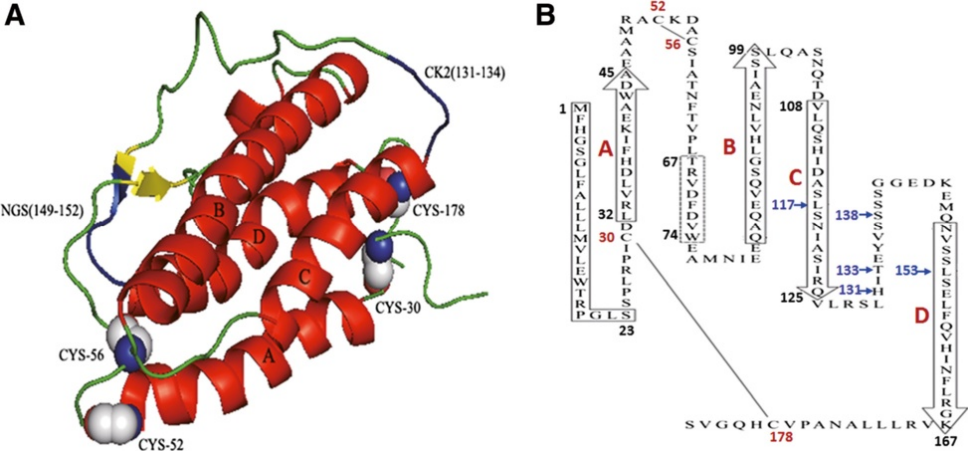
The three-dimensional structure of the *G. dobula* EPO. **a** Ribbon diagram of the predicted *G. dobula* EPO tertiary structure. The four α-helices are labeled A–D (*red*). Disulfide bonds bridge the residues 30–178 and 52–56. The important functional sites (NGS and CK2) are marked in blue. This folding pattern is strongly suggested by the large size of the two interconnecting loops AB and CD. **b** Schematic representation of the *G. dobula* EPO primary structure depicting the predicted up–up–down–down orientation of the four antiparallel α-helices (boxes with arrowheads). An apparent signal peptide sequence of 23 amino acids is delineated by the solid rectangle. The limits of each helix were drawn according to the human EPO protein sequence, as in Fig. 2. The dashed rectangle shows a predicted short region of the *β*-sheet. The locations of the two disulfide bridges are shown. Amino acid sites under positive selection (117 L, 131H, 133 T, 138S, and 153 L) are represented by blue arrows

The tertiary structures of the *G. dobula* EPOs were predicted based on a structural model of the human EPO (PDB ID code: 1BUY) [35]. The deduced amino acid sequence of the schizothoracine EPO included a signal peptide sequence predicted to be cleaved between amino acids 23 and 24; four conserved cysteine residues at 30, 52, 56 and 178 that were required for disulfide bonding and the cytokine structure; and four predicted helical domains (Fig. 4). Based on the amino acid sequence and the positions of the disulfide bonds, the *G. dobula* EPO was predicted to assume a four-antiparallel amphipathic α-helical bundle structure similar to other members of the cytokine family [37]. To compare the protein structures between high-altitude and low-altitude schizothoracine EPOs, the tertiary structure of the *S. prenanti* EPO was also predicted, producing a similar structure (Additional file 4: Figure S3).

The five sites potentially under positive selection (117 L, 131H, 133 T, 138S and 153 L) detected with the branch-site model were scattered among the third (117 L) and fourth (153 L) helices and in the loop region between the third and fourth helices (Fig. 4b).

### EPO expression profiles in three schizothoracines

**Fig. 5:**
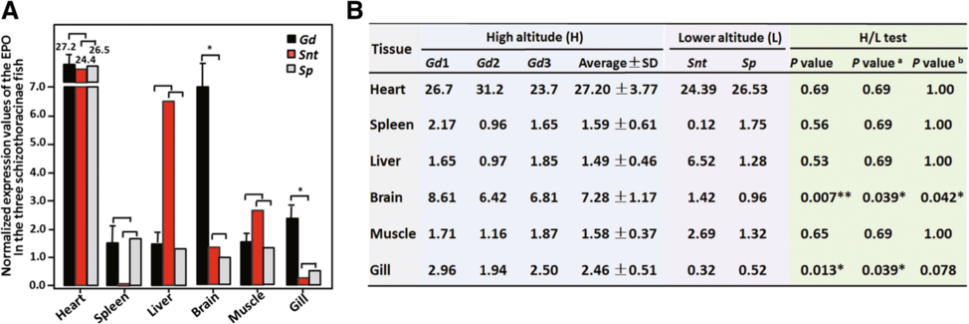
Tissue expression analysis of the EPO in the three schizothoracine fishes. **a** Six tissues (heart, spleen, liver, brain, muscle and gill) from three schizothoracine fish (*G. dobula* (*Gd*), *S. nukiangensis Tsao* (*Snt*) and *S. prenanti* (*Sp*)) were subjected to transcriptome sequencing. EPO expression levels were calculated based on normalized expression values. The gene expression levels of EPO in *G. dobula* were derived from three replicates. To compare EPO gene expression levels between the highly specialized and the non-highly specialized schizothoracines, *S. nukiangensis Tsao* and *S. prenanti* were set as the non-highly specialized schizothoracine fish group. The statistical significance was determined using a one-tailed unpaired Student’s *t*-test with *P* < 0.05. **b** Normalized expression values of the EPO genes in 6 tissues from three schizothoracine fish (*Gd*, *Snt* and *Sp*) and the relevant statistical analyses. *P* value ^a^ and *P* value ^b^ were the *P* values adjusted by FDR (false discovery rate) and Bonferroni correction, respectively

Messenger RNAs were extracted from six tissues (heart, spleen, liver, brain, muscle and gill) from *G. dobula* (three replicates), *S. nukiangensis Tsao* and *S. prenanti* and tissue-specific transcriptomes were sequenced. Sequencing and the assembly statistics are shown in Additional file 5: Table S2. To gain a further glimpse of coverage of the EPO genes in the transcriptomes, we analyzed that in the brain tissues of the three schizothoracine fishes as examples, and results were shown in Additional file 6: Table S3. In high expressing tissues like *G. doubula* brain, the number of reads for EPO ranged from 414 to 509. We then conducted comparative studies on the transcriptomes of the three species that represented the highly specialized, specialized and primitive grades of Qinghai-Tibet plateau adaptation, respectively. By comparing the normalized expression values of the EPO mRNA in each tissue, the expression profiles of the gene in six tissues of each species were obtained (Fig. 5). Significantly higher levels of EPO expression were found in the brain (*P* = 0.039 (FDR correction), *P* = 0.042 (Bonferroni correction)) and less significantly in the gill (*P* = 0.039 (FDR correction), *P* = 0.078 (Bonferroni correction)) of *G. dobula* compared with the same tissues from *S. nukiangensis Tsao* and *S. prenanti* (Fig. 5).

**Fig. 6:**
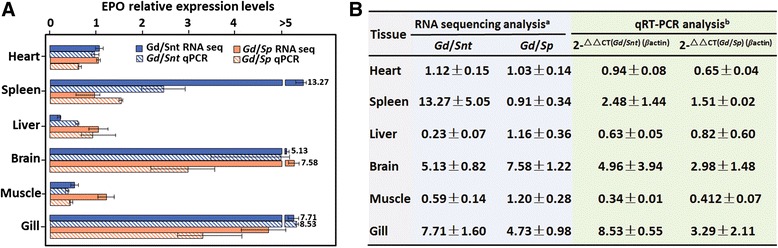
Verification of the RNA sequencing results by quantitative RT-PCR. **a** Six tissues (heart, spleen, liver, brain, muscle and gill) from *G. dobula* (*Gd*), *S. nukiangensis Tsao* (*Snt*) and *S. prenanti* (*Sp*) were subjected to quantitative RT-PCR analysis. The relative expression levels of the EPO gene deduced from the sequencing-based analysis and from qRT-PCR analysis are plotted for each tissue. **b** Ratio of EPO expression levels between the high-latitudinal species (Gd) and the two low-altitudinal (*Snt* and *Sp*) species deduced from RNA sequencing and qRT-PCR. ^a^The normalized expression values were extracted from Fig. 5b. ^b^ *β*-actin was used as an internal control in qRT-PCR analysis. qRT-PCR was performed with three biological replicates, and each sample was assayed three times. The relative expression levels between the comparison partners were calculated using the 2^−ΔΔCT^ method. Statistical significance was determined using the two-tailed unpaired Student *t*-test with *P* < 0.05

The reliability of the EPO expression profiles obtained from RNA sequencing data were verified by quantitative RT-PCR. All of the six tissues from the three species were subjected to quantitative RT-PCR analysis. Seven (Spleen *Gd*/*Snt*, Liver *Gd*/*Snt*, Brain *Gd*/*Snt*, Brain *Gd*/*Sp*, Muscle *Gd*/*Snt*, Gill *Gd*/*Snt*, and Gill *Gd*/*Sp*) out of 12 comparisons largely agreed in the direction and degree of changes between the RNA sequencing-based gene expression profiling and quantitative PCR assays (Fig. 6b). Based on these results, significantly up-regulated EPO expression levels were observed in *G. dobula* brain and gill compared with the same tissues from the other two species (Figs. 5 and 6).

### The blood Nitric Oxide (NO) levels of high- and low-altitudinal schizothoracines

We measured the concentration of nitric acid (NO) in the blood of *G. dobula* and *S. prenanti*. We found that nitric oxide (NO), a downstream signaling molecule that is inducible by EPO [38] was significantly higher in the plasma of *G. dobula* compared with the low-altitudinal *S. prenanti* (Additional file 7: Figure S4), indicating that EPO signaling was up-regulated in the high-altitude schizothoracine.

### The hypoxic adaptive feature of *G. dobula* EPO determined by cell transfection assays

**Fig. 7:**
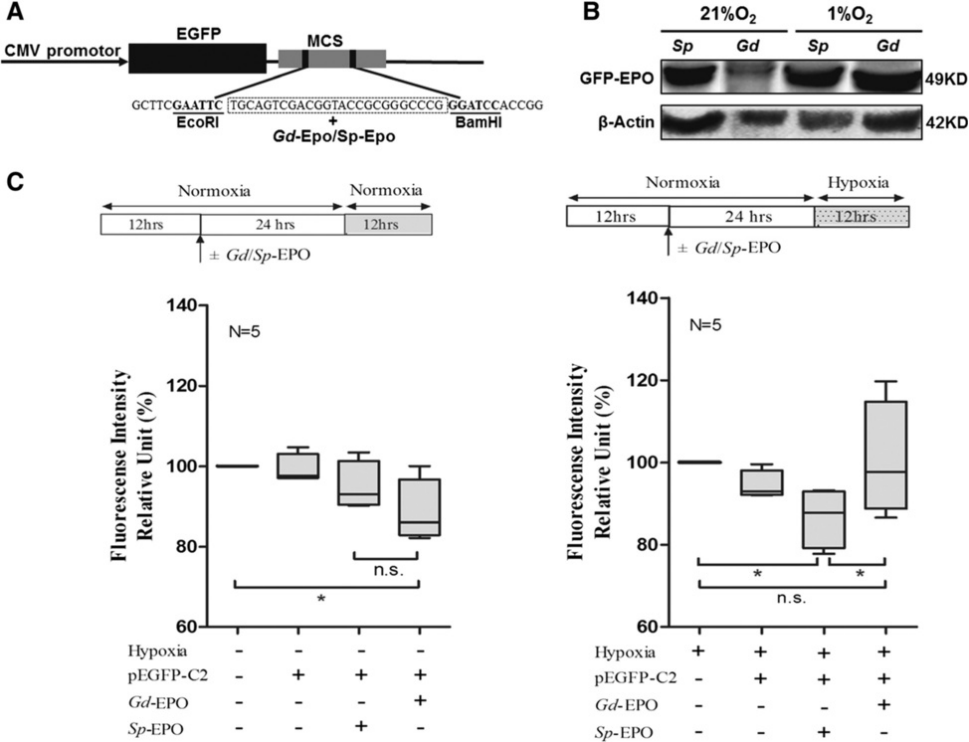
Viability analysis of 293 T cells transfected with schizothoracine EPOs under hypoxic challenge. **a** The schematic diagram of the recombinant EPO plasmid. The *G. dobula* and *S. prenanti* EPO coding sequences were cloned into the pEGFP-C2 plasmid restriction digested by BamHI and EcoRI. EPO was co-expressed with EGFP. **b** Due to the lack of proper antibodies against *G. dobula* EPO, expression of transfected EPO was determined by western blotting to detect the EGFP co-expressed with EPO. Significantly higher expression levels were detected in the cells transfected with Gd-EPO when subjected to hypoxia (<1 % O_2_). **c** The boxplots indicate relative cell viability analyses of cells transfected with schizothoracine EPOs under normoxic culture conditions (*left*) and hypoxic culture conditions (*right*). Under normoxic conditions, cells transfected with the EPO genes exhibited lower viability than the control cells (*left*). In contrast, under hypoxic conditions, the cells containing *Sp*-EPO continued to exhibit lower viability compared with the control cells, the expression of *Gd*-EPO largely reversed the reduction in viability and reached levels that were comparable to or slightly higher than the controls. Error bars represent the mean ± SE

To investigate whether *G. dobula* EPO was endowed with hypoxic protection during evolution, we cloned the *G. dobula* and *S. prenanti* EPO coding sequences into an expression vector (Fig. 7a) and transfected the plasmids into human embryonic kidney 293 T cells. Due to the lack of proper antibodies against *G. dobula* EPO, transfection efficiency was determined by the presence of the EPO mRNA and by western blotting to detect the EGFP that was co-expressed with EPO (Fig. 7b). After ensuring that similar levels of *Gd*-*EPO* and *Sp*-*EPO* were expressed in both transfections, the cells were subjected to hypoxia (<1 % O_2_) for 12 h. Cell viability was measured and compared with cells transfected with the empty vector. Cell viability is an indicator of cell health, which can be measured by examining cellular reducing conditions. When cells are alive, they maintain a reducing environment within the cytosol. Resazurin, the active ingredient of alamarBlue® reagent, is a non-toxic, cell-permeable compound that is blue in color and non-fluorescent. In viable cells, resazurin is continuously reduced to resorufin, a compound that is red in color and highly fluorescent. Using the alamarBlue measurement, we found that under normoxic conditions, the cells transfected with the EPO genes exhibited lower viability than the control cells (Fig. 7c). In contrast, under hypoxic conditions, the cells containing *Sp*-EPO continued to exhibit lower viability compared with the control cells (*P* = 0.029), while cells expressing the *Gd*-EPO largely reversed the reduction in viability and reached viability levels that were comparable to or slightly higher than the controls. These results demonstrated that there are significant functional differences between *Gd*-EPO and *Sp*-EPO, suggesting an adaptive feature of *Gd*-EPO under hypoxic conditions.

## Discussion

### Comparison of the adaptive mechanisms between Qinghai-Tibet Plateau schizothoracine fish and native Tibetans

As the world’s highest and largest plateau, the Tibetan Plateau, with an average elevation of 4500 m above sea level, imposes low oxygen concentrations on most organisms [39, 40]. Species inhabiting the Qinghai-Tibet Plateau have developed adaptive mechanisms to respond to low oxygen tension. Undoubtedly, the adaptive processes in humans native to the Qinghai-Tibet Plateau are the most abundantly studied to date [41–46]. Compared with people living at sea level, Tibetans possess several key adaptive features to cope with the hypoxic conditions. These features include decreased hemoglobin levels [43, 47], an unusually small degree of hypoxic pulmonary vasoconstriction (HPV) [47], increased levels of the vasodilator nitric oxide (NO) and increased rates of the flow of oxygen-carrying blood to tissues and of oxygen diffusion from the bloodstream into cells [48]. These adaptive features in Tibetans are possibly correlated with adaptive evolutionary changes that occurred in a few genes (i.e., EPAS1 and EGLN1) that have experienced positive selection in Tibetans [41, 43, 44].

Molecular evolutionary studies on the schizothoracine fish have been rare to date. Guan *et al*. first identified teleost-specific duplicated HIF-α genes from schizothoracine fish and suggested that HIF-1αB may be the most important regulator in the adaptation of schizothoracine fish to the aquatic environment of the Tibetan Plateau [17]. Our study is the first evolutionary study on erythropoietin (EPO), which is a downstream gene regulated by the HIFs and is the principal regulator of erythropoiesis. Elevated EPO expression in several tissues (Fig. 5) and possible positive selection sites (Fig. 3) were identified specifically in the highly specialized schizothoracine species. In combination with the enhanced cytoprotection function found with *G. dobula* EPO under hypoxic conditions, the EPO gene likely plays a role in the adaptation of the schizothoracines to the high-altitude Qinghai-Tibet Plateau. Similar to native Tibetans, higher levels of nitric oxide (NO) were detected in the plasma of *G. dobula* compared to the lower altitudinal *S. prenanti* (Additional file 7: Figure S4). Our unpublished data also showed that the blood cell number or the blood hemoglobin content of *G. dobula* is only approximately 60 or 68 % of that of *S. prenanti*, clearly suggesting that both of the blood cell number and the blood hemoglobin levels of the schizothoracine fish were reduced with the increase of elevation (data unpublished). While different genes might have experienced positive Darwinian selection in human and fish, some adaptive features such as higher NO content and lowered hemoglobin levels might have occurred in parallel as organisms adapted to the hypoxic conditions. The lowered blood cell number and hemoglobin levels in *G. dobula* suggest that factors other than EPO may also have contributed to the hypoxia adaptation of the high-altitudinal fishes.

### Sequence differences between *G. dobula* and low altitude schizothoracines might have an impact on the adaptation of EPO

In this study, we compared EPO sequences from 8 Cyprinidae fish to identify site variations and to understand the mechanism of functional evolution of the Tibetan schizothoracine EPO. Of the 8 sites that are uniquely present in the high-altitudinal species, *G. dobula* and *P. kaznakovi*, six sites (117 L, 131H, 133 T, 138S, 139S, and 153 L) are restricted to the *G. dobula* EPO, and 2 (137S and 149 N) are shared by the two highly specialized schizothoracine fish (Fig. 2). Most of these variable sites were detected as possible positively selected sites (117 L, 131H, 133 T, 138S, and 153 L) in the branch-site model (Table 2), suggesting that *G. dobula* might have experienced higher selection pressure than *P. kaznakovi*, which is a specialized schizothoracine species inhabiting comparatively lower altitudes.

The presence of 131H and 153 L in *G. dobula* resulted in a reduction of 2 CK2 phosphorylation sites (SIPE at 131–134 and SISE at 152–155) in *G. dobula* compared to the lower altitude Cyprinidae fish. Notably, more serine residues (137S, 138S, and 139S) are present in the *G. dobula* EPO (Fig. 2). Deletions in non-helical regions at the N terminus, the C terminus, and in the loops between helices resulted in the formation of EPO proteins that were readily secreted from the cell and were biologically active [49]. These regions can be ruled out as domains that are essential for function, such as the sites involved in EPO binding to its receptor. Regarding the *G. dobula* and *P. kaznakovi* EPORs, homology comparison of the proteins between the fishes and human indicated that the majority of the sites of potential positive selection are located in the ligand-binding domain and the fibronectin type III domain (Additional file 1: Figure S1 and Additional file 3: Table S1), providing evidence for potentially synergetic changes of the EPO-EPOR interacting patterns in the high-altitude fishes. However, the functional relationship between site variations in EPO and EPOR and the extent to which the amino acid variations relate to hypoxia adaptation of the EPO/EPOR remain to be clarified.

### The altered EPO expression pattern in tissues of *G. dobula*

Numerous studies have suggested that EPO is expressed in multiple tissues in different taxa [23–28]. A decrease in oxygen delivery (i.e., residence at high altitudes) stimulates EPO production, while increased oxygen delivery (i.e., erythrocytosis) decreases EPO production [50].

EPO genes have been characterized from many fish species [25–28]. The gene is clearly expressed in diverse tissues, including heart, brain, liver and spleen [51]. In the current study, significant upregulation of EPO expression in the brain and less significantly in the gill was detected in *G. dobula* compared with two other schizothoracine fish (*S. nukiangensis Tsao* and *S. prenanti*) (Fig. 5), and this was verified by qRT-PCR analysis (Fig. 6), indicating the importance of the two organs (brain and gill) in long-term hypoxia adaptation. EPO expression in the brain of *G. dobula* was approximately 3–8-fold higher than that of the lower altitude schizothoracine *S. nukiangensis Tsao* and *S. prenanti* (Fig. 6b). A medium level of EPO expression was also detected in the brain of the Plateau zokor [52]. Thus, brain tissues might be subject to extensive regulatory modification during hypoxia adaptation. Regarding the tissue EPOR expression profiling, no clear expression patterns were detected among the six tissues of the three schizothoracines (*G. dobula*, *S. nukiangensis Tsao*, and *S. prenanti*), and no significantly different expression levels of EPOR were found between the high-altitude schizothoracine (*G. dobula*) and the lower altitude schizothoracines (*S. nukiangensis Tsao* and *S. prenanti*) (detailed data not shown). Given the potential cell-protective functions of EPO against environmental stress, the upregulation of EPO expression in brain and gill might confer adaptive cytoprotective functions in the high-altitude fish.

Based on both the RNA sequencing data and qRT-PCR assays, one can conclude that EPO expression in liver and muscle of *G. dobula* was not as high as that of the low-altitudinal species, suggesting a potential down regulation of the gene in *G. dobula* in these two tissues.

Notably, hormone expression levels can vary within minutes and have very short half-lives in the tissues. Thus, although the significantly higher EPO gene expression in the brain and gills of the high-altitude species may suggest an alteration in hypoxic responses in these tissues, it is premature to conclude a causal relationship between the altered expression pattern and specific adaptive phenotypes. Similarly, the biological significance of increased EPO expression in the liver and muscle of the low-altitude species is not clear. However, the current data do indicate that EPO expression in fishes is complicated and may be shaped by multiple physiological and environmental factors, such as the altitude and temperature of the habitats. Much of the physiology of this hormone and the function it plays in environmental adaptation remains to be understood.

### The elevated cytoprotective function of *G. dobula* EPO

In addition to its erythropoietic promotion function [53], EPO has been reported to initiate adaptive cellular responses to both moderate environmental challenges and tissue damage [34, 54–57]. EPO is also subject to apoptotic stimuli in insects, strongly indicating a possible preliminary phylogenetic role for EPO in tissue protection in addition to its functional role in erythropoiesis, considering that insects lack erythropoiesis [58].

Functional analysis was performed by transfecting schizothoracine EPOs into 293 T cells. The relative cell viability for the cells transfected with the *G. dobula* EPO was significantly higher than for the cells transfected with the *S. prenanti* EPO (Fig. 7). The EPO gene in the high-altitude schizothoracine fish acquired an elevated tissue protective function under hypoxic conditions.

## Conclusions

The EPO genes of five schizothoracine fish were characterized. The schizothoracine EPO proteins exhibited the same principal domains as their mammalian counterparts. EPO expression levels were significantly different between the high-altitude plateau schizothoracine fish and low-altitude plateau schizothoracine fish, and the cytoprotective function of the EPO from the high-altitude fish was augmented during evolution. Taken together, adaptation to the Tibetan Plateau by the schizothoracine fish might have been associated with the expression of and structural changes in a cluster of genes involved in erythropoiesis and protection from hypoxia.

## Methods

### Sampling

*G. dobula* and *P. kaznakovi* fish samples were captured from Yadong, the southeast of Qinghai-Tibet Plateau and Xialaxiu, Qinghai province, respectively. *S. nukiangensis Tsao* and *S. gongshanensis* fish samples were captured from the Nujiang river and Gongshan, Yunnan Province, respectively. *S. prenanti* samples were collected from Ya’an in Sichuan Province. Permissions for capturing the fishes were obtained from the local aquaculture administration bureau of Xizhang, Qinghai, Yunnan and Sichuan province, respectively. The locations of fish sampling are listed in Table 1. At each sampling site, environmental data including elevation, latitude and longitude were documented using a handheld GPS (Explorist 210, Magellan Corp, USA).

Fishes were live trapped and anaesthetized for the entire period of surgical procedures. The sampling were conducted according to the principles expressed in the “Guide for the Care and Use of Laboratory Animals” by National Research Council of the National Academies. The animal handling and care protocol used in this study was approved by the Ethics Committee for the Use of Animal Subjects of Shanghai Ocean University. Tissue samples were immediately infused in RNAlater reagent (Sigma-Aldrich, USA), transported to the laboratory and stored at −80 °C until RNA extraction.

### Preparation of total RNA and cDNA synthesis

Total RNA was extracted from the tissue samples using TRIZOL (Invitrogen) according to the manufacturer’s protocol. The concentration of RNA was determined spectrophotometrically using a NanoDrop 2000 (Thermo). The 260/280 ratios of the samples were between 1.8 and 2.1. RNA quality was determined by observing intact rRNA on denaturing RNA gels. The extracted RNA was frozen at −80 °C for later use.

A total of 5 μg of total RNA was used for first-strand cDNA synthesis using a PrimeScript™ RT Reagent Kit with gDNA Eraser (TaKaRa) in a 20-μL volume containing 5× gDNA Eraser Buffer, 5× PrimeScript Buffer, PrimeScript RT Enzyme MIX I and RT Primer Mix. The genomic DNA elimination reaction was performed at 42 °C for 2 min, and the RNA was stored at 4 °C. The reverse-transcription reaction was incubated at 37 °C for 15 min and inactivated at 85 °C for 5 sec. The synthesized cDNA was stored at −20 °C for later use.

### Cloning and sequencing of EPO cDNA

Based on our transcriptome sequence, we designed a pair of primers (EPO_F: 5’-CCGGAATTCTTGCGAAT GTTTCACGGTTCA-3’ and EPO_R: 5’-CGCGGATCCGGCCCTTGCTCAAAATTGTCTATC-3’) to obtain the complete ORFs (open reading frames) of the EPO genes by PCR. The PCR reaction was run with an initial denaturation of 5 min, followed by 30 cycles of 30 s of denaturation at 94 °C, 30 s of annealing at 55 °C, and 60 s of extension at 72 °C. These steps were followed by a final extension of 10 min and cooling to 4 °C.

The PCR products with expected sizes were purified using the Axyprep DNA Gel Extraction Kit (Axygen Biosciences, USA), cloned into the pMD19-T vector (TaKaRa) and transformed into competent *Escherichia coli* DH5α (Tiangen). Positive colonies were identified by white/blue selection and subjected to ABI 3730 DNA sequencing with the T3 and T7 universal primers.

### Sequence analysis, multiple sequence alignment and phylogenetic analysis

The nucleotide and deduced amino acid sequences of EPOs and EPORs were compared with the sequences in the GenBank database using the BLAST program (http://blast.ncbi.nlm.nih.gov/Blast.cgi) [59]. The molecular mass and theoretical isoelectric point were predicted using the pI/Mw tool (http://www.expasy.org/tools/pi_tool.html) [60, 61]. The signal peptide was predicted using the SignalP tool (http://www.cbs.dtu.dk/services/SignalP/) [62]. The functional amino acid motifs were predicted using the MotifScan program in the PROSITE database of protein families and domains (http://prosite.expasy.org/prosite.html) [63]. The secondary sequence structure was predicted using the PSIPRED server (http://bioinf.cs.ucl.ac.uk/psipred/) [64].

Tertiary structures were modeled using both the automated and alignment modes of homology modeling provided by the SWISS-Adaptive MODEL Server (http://swissmodel.expasy.org/) [65] with the reference template of *Homo sapiens* EPO (PDB ID code:1BUY) [35]. The PyMOL tool was used for visualization and manipulation of the 3D structure.

The EPO and EPOR amino acid sequences were aligned using ClustalW using the default parameters [66]. PAL2NAL was used to transform the protein sequence alignment to coding DNA sequence alignment [67]. Nucleotide substitution model for Maximum Likelihood (ML) analysis was selected based on Akaike Information Criterion (AIC) scores using the program Modeltest 3.7, and the K2 + I model was selected as the best-fit model [68]. The phylogenetic tree was then reconstructed by employing the ML method implemented in PAUP* version 4.0b [69]. The robustness of tree topology was evaluated using 1000 bootstrap replicates.

### Testing for positive selection in *G. dobula* EPO and EPOR gene

The codeml program in PAML package version 4.8 [36] was utilized to test the presence of positive selection in EPO and EPOR protein. The free-ratio model in branch models was used to investigate the relative evolutionary rates among different branches. Branch-site models were used to determine whether lineage-specific positive selection had operated on an EPO or an EPOR branch. Tibetan Plateau schizothoracine *G. dobula* EPO was assigned to the foreground, while the other fishes, including *P. kaznakovi* (highly specialized schizothoracine), *S. nukiangensis Tsao*, *S. gongshanensis*, *S. prenanti*, *C. carp*, *C. auratus*, *D. rerio*, *E. lucius* and *C. semilaevis* were assigned as background branches. As for EPOR, the two high-altitude schizothoracines were assigned as the foreground branch, with all others as the background. The branch-site test fixes the *d*_N_/*d*_S_ > 1 category to 1 in all the branches for the null model and allows *d*_N_/*d*_S_ > 1 in the foreground branches for the alternative model (model A). The likelihood ratio test was carried out to compare the model A with a null model. The Bayes Empirical Bayes (BEB) calculation of posterior probabilities for site classes was used to calculate the probabilities of sites under positive selection [70].

### RNA sequencing and tissue expression analysis of EPOs in schizothoracines

Comparative transcriptome studies were performed between a highly specialized schizothoracine, a specialized schizothoracine and a primitive schizothoracine. Transcriptomes of six tissues (heart, spleen, liver, brain, muscle and gill) from *G. dobula*, *S. nukiangensis Tsao*, and *S. prenanti* were characterized and subjected to transcriptome sequencing projects currently being undertaken in our laboratory.

High-quality RNAs were extracted from the heart, spleen, liver, brain, muscle and gill of three individuals of each species. The cDNA fragments from different tissues were end-repaired and ligated to the proper adaptors representing distinctive indexing for each sample. For each tissue, RNA sequencing was conducted in three replicates for *G. dobula*. For *S. nukiangensis Tsao* and *S. prenanti*, RNA samples were equally pooled from the same tissues of three individuals from each species and used for pair-end library construction. The library quality and insert length were checked using the DNA High Sensitive Bioanalyzer Chip (Agilent) to ensure the proper insert size of 300–500 bps. The libraries were subjected to 100 cycles of paired-end (2 × 100 bp) sequencing on an Illumina HiSeq 1500.

FastQC (http://www.bioinformatics.babraham.ac.uk/projects/fastqc/) was used for checking the quality of the reads. The obtained raw reads were initially pre-processed by removing the adaptors and the primers using Fastx toolkit (http://hannonlab.cshl.edu/fastx_toolkit/). Low-quality reads were removed using Fastx toolkit. High-quality reads with Q30 ≥ 80 % and with a length of >50 bp were maintained. Sequence assembly was performed using Trinity software with the default parameters [71].

The Zebrafish reference genome (version GRCz10) was employed as the reference genome; then, the tissue reads were aligned to the reference genome using Tophat2 [72]. The gene count was obtained using HTSeq [73], with the results used as input for DEseq2 [74] to analyze differential expression profiles.

The gene expression levels of EPO in *G. dobula* were derived from three replicates. To compare the EPO gene expression level between the higher altitude schizothoracine and the lower altitude schizothoracine, *S. nukiangensis Tsao* and *S. prenanti* were set as the control group (the lower altitude schizothoracine fish group). Unpaired Student’s *t*-test was employed for the expression profiles comparison between the species of high altitude and low altitude using R (https://www.r-project.org/), with the significance assigned at the *P* < 0.05 level.

### Real-time RT-PCR confirmation of the EPO expression profiling among tissues

Quantitative real-time RT-PCR was used to validate the consistency of the EPO expression patterns obtained from the RNA sequencing based methodology. Real-time PCR was performed on cDNA generated from 2 μg of RNA obtained from six tissues (heart, spleen, liver, brain, muscle and gill) of three individuals each of *G. dobula*, *S. nukiangensis Tsao* and *S. prenanti*. CDNA was generated by reverse transcription using Superscript II RNase H-Reverse Transcriptase (Invitrogen) and random hexamers (50 ng/μl). The primers used to amplify EPO fragments were as follows: *Gd*/*Snt*/*Sp*-Epo-F, 5’- CTTTGCCTTACTGCTGATG -3’ and *Gd*/*Snt*/*Sp*-Epo-R, 5’- CTGGTAAAGTAATTTCTCCG -3’. Real-time PCR was performed in a 20-μL mixture containing 2× SYBR Premix Ex Taq (Til RNaseH Plus, TaKaRa), 0.4 μL of each primer (10 μM), 7.2 μL of ddH_2_O and 2 μL of cDNA on a LightCycler® 480 II (Roche). The thermocycling conditions were 95 °C for 10 min, followed by 40 cycles of 95 °C for 15 s and 60 °C for 1 min. The *β*-actin gene (*β*-actin_F: 5’-TGGCATCACACCTTCTACAACG-3’ and *β*-actin_R: 5’-AGAGGCATACAGGGACAGCACA-3’) was employed as an internal control. All of the reactions were performed with three biological replicates, and each sample was assayed three times. The relative expression levels of EPO were calculated using the 2^−ΔΔCT^ method. Statistical significance was determined using a two-tailed unpaired Student’s *t*-test with *P* < 0.05. Bonferroni and FDR (false discovery rate) corrections were conducted for *P* value correction.

### The blood Nitric Oxide (NO) levels of high- and low-altitudinal schizothoracines

Blood samples were collected from the high-altitude schizothoracine fish, *G. dobula*, and the low-altitude schizothoracine fish, *S. prenanti*, and then transferred to the laboratory within 2 days under cold conditions. Nitric oxide (NO) levels of the plasma were immediately measured. The concentrations of NO were measured by using the normal Nitrate reductase method by Nitric Oxide (NO) Assay Kit (Nanjing Jiancheng Co., Ltd., China). All measurements were performed with three biological replicates, and each sample was assayed three times. A two-tailed unpaired Student’s *t*-test was employed for the comparison between the high-altitude and low-altitude schizothoracine fish, with significance assigned at the *P* < 0.05 level.

### Transient expression of *Gd*-EPO and *Sp*-EPO in 293 T cells

The pEGFP-C2 vector was digested with enzymes as described below (New England BioLabs), and the digested products were retrieved and purified through agarose gel electrophoresis. The coding sequences of *Gd*-EPO or *Sp*-EPO was excised from the pMD19-T vector using BamHI and EcoRI and cloned into the pEGFP-C2 plasmid digested with BamHI and EcoRI. The recombinant clones were verified by sequencing. The recombinant plasmid was extracted using a QIAGEN EndoFree Plasmid Maxi Kit (Shanghai Co., Ltd., China) for 293 T cell transfection.

The human embryonic kidney 293 T cell line was cultured in Dulbecco’s modified Eagle’s medium/HIGH GLUCOSE (DMEM) (HyClone, USA) with 10 % fetal bovine serum (FBS) and penicillin-streptomycin (100 U/mL-100 μg/mL, HyClone) in a humidified environment with 5 % CO_2_ at 37 °C. The 293 T cells were seeded into 96-well culture plates (1.0 × 10^5^/mL, 100 μL) without penicillin and streptomycin. When they achieved 90 % confluence, the 293 T cells were ready for transient transfection with transfection reagents. A total of 2 μg of the plasmids pEGFP-C2, pEGFP-C2-*Gd*-EPO and pEGFP-C2-*Sp*-EPO were added to the plates in 25 μL of culture medium containing 9 μL of Entranster TM-D transfection reagent (Engreen reagent, Biosystem Co., Ltd., China). After 6 h of transient transfection, the culture medium was replaced with complete medium (supplemented with 10 % fetal bovine serum, 100 U/mL penicillin and 100 μg/mL streptomycin) and cultured for 18 h.

To examine whether the EPO mRNA was expressed in the transfected cells, total RNA was extracted and analyzed by RT-PCR. Subsequently, the transfected cells were exposed to hypoxia challenge as follows. One plate of the plasmid-transfected or control cells was subjected to hypoxia treatment for 12 h; another plate transfected with the same plasmid or control cells was maintained under normoxic conditions for the same period of time. Hypoxia exposure was controlled by the Galaxy 170R (New Brunswick, Canada), in which oxygen was replaced with nitrogen so that the final O_2_ concentration was 1 %. At least three independent replicate experiments were performed.

### Western blot analysis

Due to the lack of proper antibodies against *G. dobula* EPO, the expression of transfected EPO was determined by western blotting to detect the EGFP that was co-expressed with EPO. Proteins were extracted from cells transfected with *Sp*-EPO under normoxic conditions (21 % O_2_), cells transfected with *Gd*-EPO under normoxic conditions (21 % O_2_), cells transfected with *Sp*-EPO under hypoxic conditions (1 % O_2_), and cells transfected with *Gd*-EPO under hypoxic conditions (1 % O_2_). Equal amounts of proteins from the preparations were separated on SDS-PAGE gels and transferred onto a PVDF membrane (Millipore). The primary GFP antibody (GeneTex, GTX113617) and *β*-actin antibody (BBI, D110024) were diluted 1:5000 and 1:1000, respectively, with 1X PBST. Then, the membrane was incubated in 25 mL of secondary antibody solution (HRP-conjugated anti-rabbit IgG; Cell Signaling, 7074; 1:2000 dilution) for 1 h with slow shaking at RT. Color detection was performed with an enhanced chemiluminescence (ECL) reagent kit (Millipore). Western blot analyses for each sample were repeated at least three times.

### Viability measurement of EPO-transfected 293 T cells

Analysis of cell viability was performed using the alamarBlue® Cell Viability Reagent (Invitrogen, USA) following the manufacturer’s protocol. The reagent contains a cell-permeable blue compound that is virtually nonfluorescent but is converted to a highly fluorescent red compound by viable cells.

In this study, 10 μL of alamarBlue® (10×) was added to 96-well plates with 100 μL of DMEM/HIGH GLUCOSE (HyClone) culture medium and incubated for 20 min in a 37 °C incubator with 5 % CO_2_. The fluorescence intensity was read using a spectrophotometer at wavelengths of 560 nm for excitation and 590 nm for emission [75]. The relative cell viability graph was plotted according to the following formula: viability = (fluorescence value from transfected cells—fluorescence value of fresh DMEM) / (fluorescence value of non-transfected cells—fluorescence value of fresh DMEM) × 100. All of the measurements were performed in duplicate for at least three replicates. One-way ANOVA with a Duncan test (*P* ≤ 0.05) was performed for the fluorescence intensity from the transfected and non-transfected cells using SPSS 13.0. A *P*-value < 0.05 was considered statistically significant.

### Availability of supporting data

The EPO and EPOR gene sequences obtained in this study are available from the Genbank database under accession numbers KT188754—KT188758 and KT945255- KT945259. All other supporting data are included as additional files.

## Additional files

Additional file 1: Figure S1. ClustalW alignment of 10 EPOR amino acid sequences. The deduced amino acids of EPOR from 10 teleosts, including *G. dobula* (KT945259), *P. kaznakovi* (KT945258), *S. nukiangensis Tsao* (KT945257), *S. gongshanensis* (KT945256), *S. prenanti* (KT945255), *C. auratus* (AGN92860), *C. carpio* (BAL42514), *D. rerio* (ABB77800), *E. lucius* (XP_010873264) and *C. semilaevis* (XP_008328931) were aligned with ClustalW and used for the phylogenetic tree construction (Additional file 2: Figure S2). The EPOR ligand-binding domain and fibronectin type III (FN3) domain are marked by a bold black line and a double line, respectively. The amino acids are numbered along the right margin. The rectangular frames indicate the positively selected sites. Green shading indicates the unique amino acids that were only found in the high-altitude schizothoracines, *G. dobula* and *P. kaznakovi*. (PDF 213 kb) Additional file 2: Figure S2. Evolutionary relationships of the EPORs. Phylogenetic tree was constructed using ML method, as described in Methods. The calculated *d*_N_/*d*_S_ (ω) values (in italics) and bootstrap values are shown in each branch. The Tibetan Plateau schizothoracine *G. dobula* is highlighted by a red solid rectangle. (PDF 1514 kb) Additional file 3: Table S1. Parameter estimates for the evolutionary analysis of the schizothoracine EPOR. (DOCX 31 kb) Additional file 4: Figure S3. The three-dimensional structure of the *S. prenanti* EPO. (A) Ribbon diagram of the predicted *S. prenanti* EPO tertiary structure. The four α-helices are labeled with the letters A–D (red). Two disulfide bonds bridge residues 30–178 and 52–56. The functional sites (NGS and CK2) are marked in blue. (B) Schematic representation of the *S. prenanti* EPO primary structure depicting the predicted up–up–down–down orientation of the four antiparallel α-helices (boxes with arrowheads). An apparent signal peptide sequence of 23 amino acids is delineated by the solid rectangle. The limits of each helix are drawn according to the human EPO protein sequence, as in Fig. 2. The dashed rectangle shows a predicted short region of *β*-sheet. The locations of the two disulfide bridges are shown. (PDF 1487 kb) Additional file 5: Table S2. Overview of sequencing and assembly results. (DOCX 28 kb) Additional file 6: Table S3. Coverage estimation of EPO and EPOR genes in the brain tissues of three schizothoracine fishes. (DOCX 28 kb) Additional file 7: Figure S4. The blood Nitric Oxide (NO) levels of high- and low-altitudinal schizothoracines. (A) The blood Nitric Oxide (NO) levels of the high-altitude schizothoracine fish, *G. dobula* (*Gd*), and the low-altitude schizothoracine fish, *S. prenanti* (*Sp*), were measured using the nitrate reductase method. Significantly higher NO levels were detected in *Gd* plasma. (B) The NO levels of blood from two schizothoracine fish (*Gd* and *Sp*) and the relevant statistical analysis results. All measurements were performed with three biological replicates (*Gd*1,2,3 and *Sp*1,2,3), and each sample was assayed three times (Experiment 1,2,3). The statistical significance was determined using a two-tailed unpaired Student’s *t*-test with *P* < 0.05. (PDF 1347 kb)
