# Supplementary material for: Analysis of the erythropoietin of a Tibetan Plateau schizothoracine fish (Gymnocypris dobula) reveals enhanced cytoprotection function in hypoxic environments
Source: BMC Evol Biol. 2016 Jan 15;16:11. doi: 10.1186/s12862-015-0581-0 (PMC4714423; doi:10.1186/s12862-015-0581-0)
Supplement: Additional file 1: Figure S1. — ClustalW alignment of 10 EPOR amino acid sequences. The deduced amino acids of EPOR from 10 teleosts, including G. dobula (KT945259), P. kaznakovi (KT945258), S. nukiangensis Tsao (KT945257), S. gongshanensis (KT945256), S. prenanti (KT945255), C. auratus (AGN92860), C. carpio (BAL42514), D. rerio (ABB77800), E. lucius (XP_010873264) and C. semilaevis (XP_008328931) were aligned with ClustalW and used for the phylogenetic tree construction (Additional file 2: Figure S2). The EPOR ligand-binding domain and fibronectin type III (FN3) domain are marked by a bold black line and a double line, respectively. The amino acids are numbered along the right margin. The rectangular frames indicate the positively selected sites. Green shading indicates the unique amino acids that were only found in the high-altitude schizothoracines, G. dobula and P. kaznakovi. (PDF 213 kb) [file 12862_2015_581_MOESM1_ESM.pdf]

Figure S1. ClustalW alignment of 10 EPOR amino acid sequences

```
G. dobula               MTKMRSNRLKIVF-MLCLAS--VTTGGQYFESKVAQLLQDETEDIKCFVEE-KDLTCFWE 56
P. kaznakovi            MTKMRSNRLKIVF-MLCLAF--VTTGGQYFESKVAQLLQDETEDIKCFVEE-KDLTCFWE 56
S. gongshanensis        MTKMRSDRLKIVF-MLCLAF--VTTGGQHFESKVAQLLQDETEDIKCFVEG-KDLTCFWE 56
S. nukiangensis Tsao    MTKMRSDRLKIVF-MLCLAF--VTTGGQHFESKVAQLLQDETEDIKCFVEG-KDLTCFWE 56
S. prenati              MTKMRSDRLKIVF-MLCLAF--VTTGGQHFESKVAQLLQDETEDITCFVEG-KDLTCFWE 56
D. rerio                MTKMRSDRLKIVF-MLCLAF--VTSGGKYFESKVAQLIRDETEDIKCFVEGKKDFTCFWE 57
C. auratus              MTKMRSNRLKIIF-MLCLAC--VTTGGQHFESKVAQLLQDETEHIKCFVEG-KDLTCFWE 56
C. carpio               MTKM-SDRLKIVF-MLCVAF--VTTGGQHFESKVAQLLQDETEDIKCFVEL-KDLTCFWE 55
C. semilaevis           MNAAHRNRAAALYALLCVCV--SVLGGKTWEEKFSIMRREDPENPKCFAEDRMNLTCFWE 58
E. lucius               MTNDHLKKLLVYC-MLCCTQKIIVQGAQTFQTKVALLRHVEPNNPKCFAEGIRDLTCFWE 59
                        *.    .:      :**      . *.: :: *.: : : : :. .**.*   ::*****

G. dobula               DEEERNNSHDQYTFTYSFENKNKMACAVSSLSLLASNKSVLFGILPKPPLFMTINVQVLR 116
P. kaznakovi            DEEERNNSHDQYTFTYSFENKNKMACAVSSLSLLASNKSVLFSILPKPPLFTTINVQVLR 116
S. gongshanensis        EEEERNNLHDQYTLTYSYENKNKMAYAVSSLSLLASNKTVLFCKLPKPPFFTTLDVQVLH 116
S. nukiangensis Tsao    EEEERNNLHDQYTLTYSYENKNKMAYAVSSLSLLASNKTVLFCKLPKPPFFTTLDVQVLH 116
S. prenati              EEEERNNLHDQYTLTYSYENKNKMAYAVSSLSLLASNKTVLFCKLPKPPFFTTLDVQVLH 116
D. rerio                KEDGTNYSQDNYTFTYTYMNENKMDCAVSSLFLLSSNRSVFFCKLPKALFFTSLDVQVLR 117
C. auratus              EEEERNHLHNQYTFTYSYENKNKMACAVSSLSLLASNKTIFFCKLAKTPFFTTLHVQVHR 116
C. carpio               EEEERNDL--QYTFTYSYENKNKMACVVSSLSLLASNKTVFFCKLPKTPFFTTLDVQVFR 113
C. semilaevis           EDEETAGPVEEYSFTYNYQHEDSVQCPLRSLL-SSDRRXXXXXXXXXXXMFVQMDIYVHR 117
E. lucius               EDEERAGSAKQYSFMYTYQNENSSECNVTALP-AAGGRILYFCRLSQTQLFVPLDIQVFR 118
                        FN3 super family domain (EPOR ligand binding domain)
                        .::       :*:: *.: .::.    :  :*   :  :           :*  :.: * :

G. dobula               DGRRLYSRSLNAENLYFLDPPRNLTVMSSGKEGQLNVSWWPPPVKYLYDSVIYEVRYAVE 176
P. kaznakovi            DGRRLYSRSLNAENLYFLDPPRNLTVMSSGKEGQMNVSWWPPPVKYLYDSVIYEVRYAVE 176
S. gongshanensis        DGRMLYSRRLNAENFLFLDPPRNLTVMSSGKEGQLNVSWLPPPLKYMDDSMIYEVRYAVE 176
S. nukiangensis Tsao    DGRMLYSRRLNAENFLFLDPPRNLTVMSSGKEGQLNVSWLPPPLKYMDDSMIYEVRYAVE 176
S. prenati              DGRMLYSRRLNAENFLFLDPPRNLTVMSSGKEGQLNVSWLPPPLKYMDDSMIYEVRYAVE 176
D. rerio                DGQMLYNRSLNVENILLTDPPRNVTVWSSGKEGQLNVSWLPPAVKYIDDSLIYEVRYAVE 177
C. auratus              DGQKLYTRSLNAENFLFLDPPRNLTVLSSGKEGQLNVSWLPPQLKYMDDSMIYEVRYAAE 176
C. carpio               DGRMLYSRSLNAENYLFLDPPRNLTVLSSGKEGQLNVSWLPPLLKYMDDSMIYEVRYAVE 173
C. semilaevis           KGGLIHNRTLFVDMVFLLDPPANVTVNRTEKQDQLNVSWVAPKLKYMDDSMMYQIRYSSA 177
E. lucius               DGLLIHNRRLHIELCFLLDPPVNLTVKRTEKQGQLKASWMPPPLKYLDDSMIYEVSYVIA 178
                                                                  FN3 domain
                        .*  ::.* *  :   : *** *:**  : *: *::.**  * :**: **::*:: *

G. dobula               GSNMGKVEVIKVSTMLVLRGLQSDTRYKVWIRTKPDGVSYKGYWSAWTEPVFGVTPPSDV 236
P. kaznakovi            GSNMGKVEVIKVSTMLVLRGLQSDTRYKVWIRTKPDGVSYKGYWSAWTEPVFGVTPPSDV 236
S. gongshanensis        GSNMGKVEVMKVSTMLVLLGLQSDTRYKVWVRAKPDGVSYKGYWSAWTEPVFGVTPPSDM 236
S. nukiangensis Tsao    GSNMGKVEVMKVSTRLVLLGLQSDTRYKVWVRAKPDGVSYKGYWSAWTEPVFGVTPPSDM 236
S. prenati              GSNMGKVEVMKVSTMLVLLGLQSDTRYKVWVRAKPDGVSYKGYWSAWTEPVFGVTPPSDV 236
D. rerio                DSHMGKVEETKASTMLVLRGLQPDTRYKVWVRVKPDGVSYKGYWSSWTSPVIAVTPPGSM 237
C. auratus              GSDLGKVEVIKVSTMLVLLGLESDTRYKVWVRVKPDGVAYKGYWSAWTEPVFGVTPPSDV 236
C. carpio               GSNMGKVEVIKVSTMLVLLGLQSDTRYKVWVRVKPDGVAYKGYWSAWTEPVLGVTQPSDL 233
C. semilaevis           DGLETQVTESLAVTEKILTNLRPGVKYEVQVRIKLDGISYDGYWSAWSTGVFMETPPAEF 237
E. lucius               GTQMGQVEEVQASSEVILLGLQPGAKYSVRIRVKLDGVSYNGYWSAWTDPVLMATLPGDL 238
                        FN3 domain
                             :*    . :  :*  *.  .:*.* :* * **::*.****:*:  *:  * *...

G. dobula               DPLIVLLALFIVLILCLLSLTGFLSQHKFLLKKLWPDIPTPEHKFPGLFSVYKGDFKEWM 296
P. kaznakovi            DPLIVLLVLFIVLILCLLSLTVFLSQHKFLLKKLWPDIPTPEHKFPGLFTVYKGDFKEWM 296
S. gongshanensis        DPLIVLLVLFIGLILCLLTLTVFLSQHKFLLKKLWPDIPTPEHKFPGLFTVYKGDFKEWM 296
S. nukiangensis Tsao    DPLIVLLVLFIGLILCLLTLTVFLSQHKFLLKKLWPDIPTPEHKFPGLFTVYKGDFKEWM 296
S. prenati              DPLIVLLVLFIGLILCLLTLTVFLSQHKFLLKKLWPDIPTPEHKFPGLFTVYKGDFKEWM 296
D. rerio                DPLIVLLVVFIILILCLLSMTVILSHHKFLLKKLWPDIPTPEHKFPGLFTVYKGDFKEWM 297
C. auratus              DPLIVLLVLSIGLILCLLSLTVILSHQKFLLKKLWPDIPTPEHKFPGLFTVYKGDFKEWM 296
C. carpio               DPLIMVLVLFIGLILCLLSMTVILSHHKFLLKKLWPDIPTPEHKFPGLFTVYKGDFKEWM 293
C. semilaevis           DPLILCLTLIISFILLVFSLTILLSHRRFLTKKIWPVVPTPENKFPGLFSVYGGDFQEWL 297
E. lucius               DPLIVSLTLIISLILTLLSLTVLLSHRRFLLNKIWPVIPNPESKFQGLFTVYGGDFEEWL 298
                        ****: *.: * :** ::::* :**:::** :*:** :*.** ** ***:** ***:**:
```

```
G.dobula              SQNNGYMWGRSVHVYTEELLSPLEVLSEVSLT----SHGTSQREERKPAEEEEEKKPAEE 352
P.kaznakovi           SQNNGNMWGRSVHVYTEELLSPLEVLSEVSLT----SHGTSQREERKPAEEEE------- 345
S.gongshanensis       SQNNGNMWGRSVHVYTEELPSPLEVLSEVSLT----SHGTSQREERKPAEEEE------- 345
S.nukiangensis Tsao   SQNNGNMWGRSVHVYTEELPSPLEVLSEVSLT----SHGTSQREERKPAEEEE------- 345
S.prenati             SQNNGNMWGRSVHVYTEELPSPLEVLSEVSLT----SHGTSQREERKPAEEEE------- 345
D.rerio               SQNSGSMWARSVQMYTEELPSPLEVLSEVSLS----P-----LDERKLVR---------- 338
C.auratus             SHNNGSMWGRSVHVYTEELPSPLEVLSEVSLT----SHDTSHREEVKPAEEDE------- 345
C.carpio              SQNNSSMWGRSVQVYTEELPSPLEVLSEVSLT----SHGTSQREERKPVEEDE------- 342
C.semilaevis          GHTRGGLWVAADYLFSEERPSLLEVISEFRLGPPLVSPPLPPKAPRGLTLGREE-----N 352
E.lucius              GYSAGGLCLRPAYFYSEELPAPLEVLSEVSLDLP--SSPLPPKAKQAPGED--------- 347
                      . . . :      .::**  : ***:**. *

G.dobula              ERESQRSDSGLTDRWREPPQAHWLMEQLRALQENPESRPQSALLQSHDTYVTLNQ---GD 409
P.kaznakovi           ERESERSDSGLTDRWREPPQAHWLMEQLRALQENPESRPQSSLLQSHDTYVTLNQ---GD 402
S.gongshanensis       ERESERSDSGLTDRWQEPPQVHWLMEQLRHLQENPESRSQSSILQSHDTYVTLNQ---GD 402
S.nukiangensis Tsao   ERESERSDSGLTDRWREPPQAHWLMEQLRHLQENPESRSQSSILQSHDTYVTLNQ---GD 402
S.prenati             ERESERSDSGLTDRWREPPQAHWLMEQLRHLQENPESRSQSSILQSHDTYVTLNQ---GD 402
D.rerio               -DEDQRSDSGLTE------PPHWLMEQLRALQENPESLSRSTLLQSHDTYITLNH---SS 388
C.auratus             EKESERSDSGLTDRWPDPPHAHWLMDQLRALQENPESHSQSSLLQSHDTYVTLNQ---GD 402
C.carpio              EKESERSDSGLTDRWREPPQAHWLMEQLRALQENPQSLSQSLLVQSHDTYVTLNQNSQGD 402
C.semilaevis          QEVLERSASTVT-------HDPWLMDRV---------LHQTALLESQDPYVTLSN---PP 393
E.lucius              -EKLKRID-SLMERWRETPHEHWLMDQLRVLHQLPGP--QCSQLESQDAYVTLNAQN-HS 402
                          :*    :           ***:::           :   ::*:* *:**.

G.dobula              IEQQVDDVFEETLPLQTLFSTAGTSSLSTSHSDLGSLLQSSGSGRLSSQSSFEYPNHTWP 469
P.kaznakovi           IEQQVDDVFEETLPLQTLFSTAGTSSLSTSHSDLGSLLQSSGSGRLSSQSSFEYPNHTWP 462
S.gongshanensis       NEQQVDDVFEETLPLQTLFTTAGTSSLSTSHSDLGSLQQSSGSGRLSSQSSFEYPNHTWP 462
S.nukiangensis Tsao   NEQQVDDVFEETLPLQTLFTTAGTSSLSTSHSDLGSLQQSSGSGRLSSQSSFEYPNHTWP 462
S.prenati             NEQQVDDVFEETLPLQTLFTTAGTSSLSTSHSDLGSLQQSSGSGRLSSQSSFEYPNHTWP 462
D.rerio               GGQREDDVFEETLPLQTLFTSAGTSSLNASHSDLGSLRQSSASGRLSSQSSFEDPNHPWP 448
C.auratus             IEQQADDVFEETLPLQTLFSSAGTPSLSISHSDLGSLQQSSGSGRLSSQSSLEYPNHTWP 462
C.carpio              GEREVDDVFEETLPLQTLFTTTGTTSLSTSHSDLGSLQQSSGSGRLSSQSSFEYPNQTWP 462
C.semilaevis          DGEQVDELLEESLPLEVLFHP-GVTALCESHSDLGSGQQSSGS-RMSSQSSFEYPNQAWT 451
E.lucius              GEEPLDDILEETLPLQALFASGRT--CSESHSDLGSLQQSSGSGRLSTQSSFEYPNHTWP 460
                        .  *:::**:***:.**    .     *******  ***.* *:*:***:* **: *

G.dobula              PKGPGYAYMAVADSGVSMEYSPMSSSRIAEVGRHSMYTNDYKNEIV--QWPLSGQYVKSG 527
P.kaznakovi           PKGPGYAYMAVADSGVSMEYSPMSSSRIAELGRHSMYTNDYKNEIV--QWPLSGQYVKSG 520
S.gongshanensis       PKGPGYAYMAVADSGVSMDYSPMSSSKIAELGRHRMYTNDYKNEMFLHKWPLSGQYVKSG 522
S.nukiangensis Tsao   PKGPGYAYMAVADSGVSMDYSPMSSSKIAELGRHRMYTNDYKNEMFLHKWPLSGQYVKSG 522
S.prenati             PKGPGYAYMAVADSGVSMDYSPMSSSKITELGRHRMYTNDYKNEIFLHNWPLSGQYVKSG 522
D.rerio               PKGPGYAYMAVADSGVSMDYSPMSSSRIIEIGKRSFYANEYKNEIFGYKWPFSAQQVQSG 508
C.auratus             PKGPGYAYMAVADSGVSMDYSPMSSSRIAELGRHRIYTNDYKNEIFPHKWPLSGQYIKSG 522
C.carpio              PKGPGYTYMAVADSGVSMDYSPMSSSRITELGRHRMYTNDYKNEIFLHKWPLFGEHNKSG 522
C.semilaevis          RKGPGYTYMAVADSGVSMDYSPMS--RVEDVGRVPVYANDYKNDIMSQKTLFLQRQRAVD 509
E.lucius              PKGPGYTYMAVADSGVSMDYSPMSSSRIDYMGKGVIYTNEYRNEIPAHRQPIPFAF---- 516
                       *****:***********:*****  ::  :*:  .*:*:*:*::   .  :

G.dobula              SESYTGAVW 536
P.kaznakovi           SESYTGAVW 529
S.gongshanensis       SESCTGPVW 531
S.nukiangensis Tsao   SESCTGPVW 531
S.prenati             SESCTGPVW 531
D.rerio               Y-------- 509
C.auratus             --------- 522
C.carpio              SESCMGPVW 531
C.semilaevis          DND------ 512
E.lucius              --------- 516
```
