# Supplementary figures and images for: Analysis of the erythropoietin of a Tibetan Plateau schizothoracine fish (Gymnocypris dobula) reveals enhanced cytoprotection function in hypoxic environments
Source: BMC Evol Biol. 2016 Jan 15;16:11. doi: 10.1186/s12862-015-0581-0 (PMC4714423; doi:10.1186/s12862-015-0581-0)

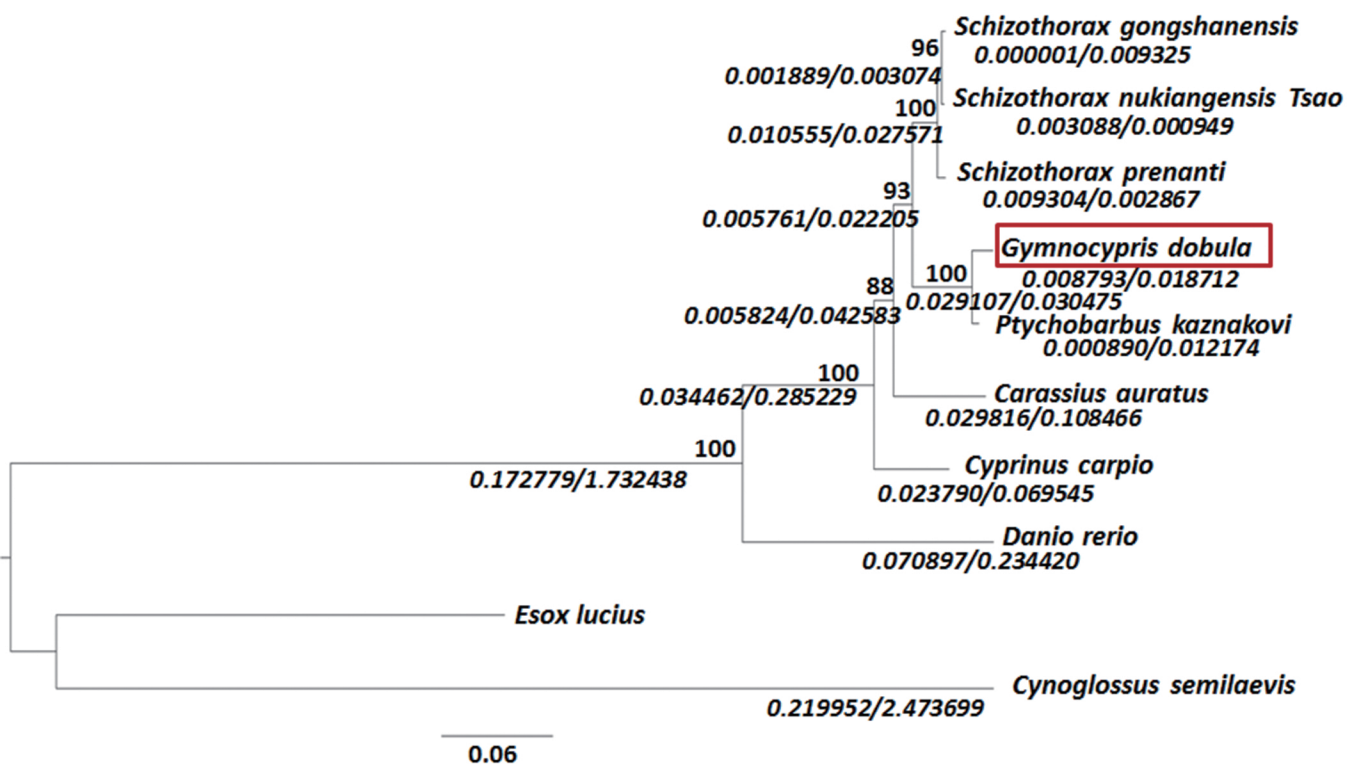

Supplement: Additional file 2: Figure S2. — Evolutionary relationships of the EPORs. Phylogenetic tree was constructed using ML method, as described in Methods. The calculated d N/d S (ω) values (in italics) and bootstrap values are shown in each branch. The Tibetan Plateau schizothoracine G. dobula is highlighted by a red solid rectangle. (PDF 1514 kb) [file 12862_2015_581_MOESM2_ESM.pdf]

**A**

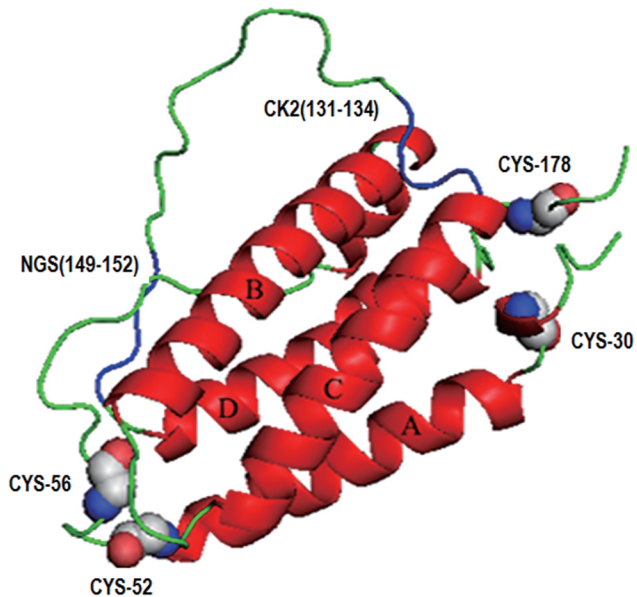

# B

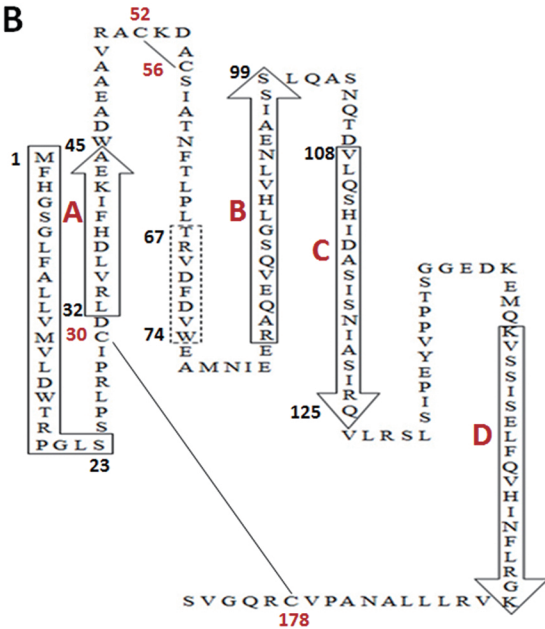

Supplement: Additional file 4: Figure S3. — The three-dimensional structure of the S. prenanti EPO. (A) Ribbon diagram of the predicted S. prenanti EPO tertiary structure. The four α-helices are labeled with the letters A–D (red). Two disulfide bonds bridge residues 30–178 and 52–56. The functional sites (NGS and CK2) are marked in blue. (B) Schematic representation of the S. prenanti EPO primary structure depicting the predicted up–up–down–down orientation of the four antiparallel α-helices (boxes with arrowheads). An apparent signal peptide sequence of 23 amino acids is delineated by the solid rectangle. The limits of each helix are drawn according to the human EPO protein sequence, as in Fig. 2. The dashed rectangle shows a predicted short region of β-sheet. The locations of the two disulfide bridges are shown. (PDF 1487 kb) [file 12862_2015_581_MOESM4_ESM.pdf]
