# Supplementary material for: Analysis of the erythropoietin of a Tibetan Plateau schizothoracine fish (Gymnocypris dobula) reveals enhanced cytoprotection function in hypoxic environments
Source: BMC Evol Biol. 2016 Jan 15;16:11. doi: 10.1186/s12862-015-0581-0 (PMC4714423; doi:10.1186/s12862-015-0581-0)
Supplement: Additional file 3: Table S1. — Parameter estimates for the evolutionary analysis of the schizothoracine EPOR. (DOCX 31 kb) [file 12862_2015_581_MOESM3_ESM.docx]

**Table S1.** Parameter estimates for the evolutionary analysis of the schizothoracine EPOR

Models Estimate of parameters *ℓ* Positively selected sites

**Branch model:**

**free-ratio** (see Supplemental Fig. S2 for ω values for each node) -6564.86 None

**Branch-site models (LRT for branch-site: 2.78, *P*-value=0.096 with df=1)**

Model Null *p*_0_=0.41, *p*_1_= 0.09, (*p*_2_+*p*_3_=0.50), ω_0_=0.12, ω_1_=1.00, ω_3_=1.00 -6526.25 None

Model A *p*_0_=0.70, *p*_1_=0.15, (*p*_2_+*p*_3_=0.15), ω_0_=0.12, ω_1_=1.00, ω_3_=4.84 -6524.86 57D (*p*=0.809), 64S (*p*=0.593), 74F(*p*=0.837), 106L (*p*=0.782),

110I (*p*=0.821), 111N (*p*=0.825), 120R (*p*=0.748), 132Y (*p*=0.993),

156W (*p*=0.927), 160V (*p*=0.829), 161L (*p*=0.781), 164Y (*p*=0.847)

167V (*p*=0.815), 207I (*p*=0.805), 209T (*p*=0.769), 316L (*p*=0.847),

447L (*p*=0.834), 488E (*p*=0.827), 516Q (*p*=0.751)

**Note:** The branch-site model in codeml program divides all the sites into four classes, class 0, 1, 2, and 3. Class 0 is for the sites under purifying selection. Class 1 is for the sites under neutral selection. Class 2 and 3 are for the sites that have positive selection in foreground branches. In this table, *p*_0_ and ω_0_ stand for the percentage and averaged omega value of class 0 sites in the alignment. *P*_1_ and ω_1_ stand for the percentage and averaged omega value of class 1 sites. *P*_2_+*p*_3_ and ω_2_+ω_3_ are for the percentage and averaged omega value of the sites in class 2 and 3.
