# Supplementary material for: Analysis of the erythropoietin of a Tibetan Plateau schizothoracine fish (Gymnocypris dobula) reveals enhanced cytoprotection function in hypoxic environments
Source: BMC Evol Biol. 2016 Jan 15;16:11. doi: 10.1186/s12862-015-0581-0 (PMC4714423; doi:10.1186/s12862-015-0581-0)
Supplement: Additional file 5: Table S2. — Overview of sequencing and assembly results. (DOCX 28 kb) [file 12862_2015_581_MOESM5_ESM.docx]

**Table S2** Overview of sequencing and assembly results

Sequencing results Assembly results

Species

Raw bases Clean reads Clean bases Q30 GC content Contig N50 Minimal length Mean length Max length

(G) Number (G) (%) (%) Number (bp) (bp) (bp) (bp)

*G. dobula*_1 94 206,939,304 54 80 45 176,206 2,561 201 1,232 28,668

*G. dobula*_2 91 244,277,066 63 80 46 182,829 2,644 201 1,323 43,715

*G. dobula*_3 92 247,656,921 64 80 47 138,918 2,827 201 1,441 27,178

*S. nukiangensis* 81 226,322,373 55 80 47 578,978 1,907 101 660 18,071

*S. prenanti* 73 172,074,980 38 80 46 164,077 2,791 201 1346 42,708
