# Supplementary material for: Analysis of the erythropoietin of a Tibetan Plateau schizothoracine fish (Gymnocypris dobula) reveals enhanced cytoprotection function in hypoxic environments
Source: BMC Evol Biol. 2016 Jan 15;16:11. doi: 10.1186/s12862-015-0581-0 (PMC4714423; doi:10.1186/s12862-015-0581-0)
Supplement: Additional file 6: Table S3. — Coverage estimation of EPO and EPOR genes in the brain tissues of three schizothoracine fishes. (DOCX 28 kb) [file 12862_2015_581_MOESM6_ESM.docx]

**Table S3**  Coverage estimation of EPO and EPOR genes in the brain tissues of three schizothoracine fishes

Gene Maximum length Effective length Number of reads

Species (bp) (bp)

***EPO***

*G. dobula_*1 3242 3079 414

*G. dobula*_2 2150 1984 509

*G. dobula*_3 3204 3039 507

*S. nukiangensis* 966 804 101

*S. prenanti* 1955 1772 21

***EPOR***

*G. dobula_*1 4283 4120 130

*G. dobula*_2 3172 3006 1878

*G. dobula*_3 590 425 128

*S. nukiangensis* 3242 3080 104

*S. prenanti* 1813 1630 5
