# Supplementary material for: Analysis of the erythropoietin of a Tibetan Plateau schizothoracine fish (Gymnocypris dobula) reveals enhanced cytoprotection function in hypoxic environments
Source: BMC Evol Biol. 2016 Jan 15;16:11. doi: 10.1186/s12862-015-0581-0 (PMC4714423; doi:10.1186/s12862-015-0581-0)
Supplement: Additional file 7: Figure S4. — The blood Nitric Oxide (NO) levels of high- and low-altitudinal schizothoracines. (A) The blood Nitric Oxide (NO) levels of the high-altitude schizothoracine fish, G. dobula (Gd), and the low-altitude schizothoracine fish, S. prenanti (Sp), were measured using the nitrate reductase method. Significantly higher NO levels were detected in Gd plasma. (B) The NO levels of blood from two schizothoracine fish (Gd and Sp) and the relevant statistical analysis results. All measurements were performed with three biological replicates (Gd1,2,3 and Sp1,2,3), and each sample was assayed three times (Experiment 1,2,3). The statistical significance was determined using a two-tailed unpaired Student’s t-test with P < 0.05. (PDF 1347 kb) [file 12862_2015_581_MOESM7_ESM.pdf]

A

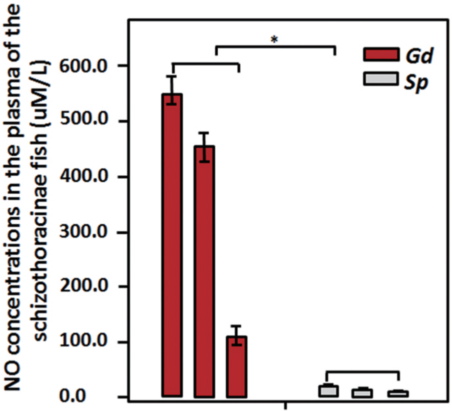

B

Species Individual (1,2,3)	Experiment Repeat			Average ± SD uM/L	*Gd*/*Sp* *P* value
	Experiment 1	Experiment 2	Experiment 3		
*Gd*1	534.83	539.68	585.82	553.44 ± 28.14	
*Gd*2	466.57	426.84	474.83	456.08 ± 25.66	
*Gd*3	130.80	89.87	111.36	110.68 ± 20.47	*$P<0.05$
*Sp*1	20.20	18.63	21.60	20.14 ± 1.49	
*Sp*2	18.23	16.04	19.78	18.02 ± 1.88	
*Sp*3	9.30	5.13	11.21	8.55 ± 3.11	
